# Traffic-Related Air Pollution and Respiratory Symptoms in Children Living along Trunk Roads in Chiba Prefecture, Japan

**DOI:** 10.2188/jea.13.108

**Published:** 2007-11-30

**Authors:** Masayuki Shima, Yoshio Nitta, Motoaki Adachi

**Affiliations:** 1 Department of Public Health, Graduate School of Medicine, Chiba University.

**Keywords:** air pollution, automobile exhaust, children, cohort study, asthma

## Abstract

Automobile exhaust is considered to be a potential risk factor for respiratory diseases. To investigate the effects of traffic-related air pollution on respiratory symptoms among children who lived near trunk roads, we conducted a cohort study on 2,506 schoolchildren in eight different communities in Japan. Over that four-year period, the prevalence of asthma was higher among girls who lived less than 50 m from trunk roads (roadside areas) than among girls in the other areas studied. Testing for trends showed that the prevalence of asthma among girls increased significantly with increases in the concentration of air pollution in each area. Among boys, the prevalence of asthma did not differ in relation to the distance from roads, although the rate was higher in urban areas than in rural areas. The incidence of asthma during the follow-up period significantly increased among boys living in roadside areas relative to rural areas (odds ratio = 3.75; 95% confidence interval: 1.00-14.06). Among girls, the incidence of asthma also increased (odds ratio = 4.06; 95% confidence interval:0.91-18.10), although the risk was not significant. These findings suggest that traffic-related air pollution may be of particular importance in the development of asthma among children living near major trunk roads with heavy traffic.

Recently, the increasing automobile traffic in Japan has caused considerable increases in levels of air pollution derived primarily from automobile exhaust.^1^ In areas adjacent to trunk roads, the concentrations of atmospheric nitrogen dioxide (NO_2_) and suspended particulate matter (SPM) are higher than in the general environment.^2^^,^^3^ The potential effect of these concentrations on the health of residents who live near trunk roads is a matter of concern.^3^^-^^6^

The number of diesel-powered vehicles has been increasing in European countries and Japan. Exposure to high levels of diesel exhaust has been associated with low pulmonary function and asthma among bus-garage and railroad workers.^7^^,^^8^ Also, NO_2_ at high concentrations is known to increase bronchial responsiveness in patients with asthma.^9^ Exposure to ambient air pollution has been reported to aggravate symptoms in children with asthma.^10^^,^^11^ Some studies have also shown high prevalence rates of asthma in areas with high concentrations of air pollution.^12^^-^^14^ However, the available epidemiologic evidence is limited and the effect of ambient air pollution on respiratory symptoms is not conclusive.^15^^,^^16^ Most of the previous epidemiologic studies were cross-sectional, but a prospective cohort study should be valuable in estimating the effect of air pollution on respiratory symptoms.^17^

To evaluate the association between air pollution from automobile traffic and respiratory health, children who live in areas that contain trunk roads were examined repeatedly over a period of four years for respiratory symptoms. We investigated the association of the prevalence and incidence of respiratory symptoms in children with the distance of their homes from trunk roads.

## METHODS

**Figure 1.**
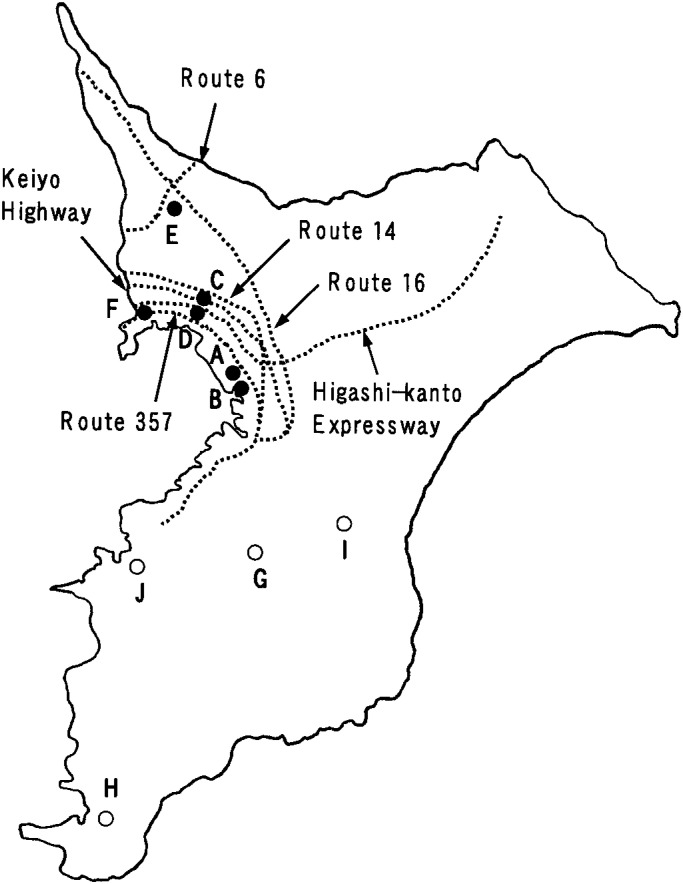
Location of study schools and major trunk roads in Chiba Prefecture, Japan. A-F schools (closed circles) are located in urban areas, and G-J schools (open circles) are in rural areas. The dotted lines show major trunk roads.

The study subjects were entire first to third grade children (n = 3,234, aged 6-9 years) as of September 1992 who attended ten elementary schools from eight different communities in Chiba Prefecture, Japan. The locations of the schools are shown in Figure 1. Of these schools, six in four communities (Chiba [A, B], Funabashi [C, D], Kashiwa [E], and Ichikawa [F]) are located in urban areas, and their school districts are intersected by major trunk roads that are all national highways or motorways. The daytime average traffic density of these roads ranged from about 37,000 to 83,000 vehicles every 12 hours in 1997; heavy vehicles accounted for about 18 to 44% of the traffic at that time (Table 1). The subjects in Chiba and Funabashi were pupils from two schools in adjacent districts in each community. The other four schools are in rural communities (Ichihara [G], Tateyama [H], Mobara [I], and Kisarazu [J]). In each of these cases, there were no major roads or factories within the school district.

**Table 1.** The daytime average traffic density of the trunk roads in urban school districts.

City	Trunk road	1990		1997	
	
		Traffic density (vehicles/12hours)	Heavy vehicles (%)	Traffic density (vehicles/12hours)	Heavy vehicles (%)
Chiba	Route 14	46,702	20.1	41,000	23.5
	Route 16	46,347	23.6	49,176	25.7

Funabashi	Keiyo Highway	76,752	21.8	71,358	20.5
	Higashi-kanto Expressway	71,645	34.3	76,002	33.5
	Route 357	33,775	35.2	36,512	37.4

Kashiwa	Route 6	43,305	15.3	39,812	17.6
	Route 16	44,883	28.3	39,091	33.4

Ichikawa	Keiyo Highway	82,180	22.5	83,097	18.9
	Higashi-kanto Expressway	59,541	33.8	63,732	32.3
	Route 357	52,823	39.7	53,397	43.8

The average of the concentrations of air pollutants for the 5 years from 1991 to 1995 measured at ambient air monitoring stations in the vicinities of these schools are shown in Table 2. The greatest distance between a school and the monitoring station was 3 km for one rural school, whereas the distances were 0-1.4 km for the other nine schools. For all pollutants, the concentrations were higher in urban than in rural communities. In the roadside areas in urban communities, the concentrations of NO_2_ and SPM were considerably higher than in the general environment.

**Table 2.** Average concentrations* of atmospheric air pollutants in the study communities.

City	Nitrogen dioxide (ppb)		Sulfur dioxide (ppb)		Suspended particulate matter (mg/m^3^)	
		
	General environment^†^	Roadside^‡^	General environment^†^	Roadside^‡^	General environment^†^	Roadside^‡^
Chiba	25.4	38.2	7.6	11.2	52.0	64.0
Funabashi	29.6	38.2	6.8	-	53.2	54.6
Kashiwa	26.0	38.2	6.8	-	51.8	53.2
Ichikawa	32.0	39.4	7.4	-	50.4	-
Ichihara	10.6	-	4.2	-	33.4	-
Tateyama	7.2	-	3.6	-	28.2	-
Mobara	15.2	-	-	-	37.8	-
Kisarazu	19.8	-	4.6	-	42.2	-

* Average of annual concentrations of air pollutants for the period 1991-1995.

^†^ Data from general environment monitoring stations located in near the study school.

^‡^ Data from automobile exhaust monitoring stations located along trunk roads near the study school.

A survey of respiratory symptoms was conducted on all subjects in September or October of each year from 1992 through 1995, with the consent of principals of the participating schools. In the first and fourth surveys, a standard respiratory symptom questionnaire, the modified Japanese version of ATS-DLD-78-C,^18^ was sent through the schools to the parents of all subjects. The questionnaire covered the respiratory symptoms and medical history of the children, feeding method in infancy, history of allergic diseases of the parents, smoking habits of the household members, structure of the house, and type of heating. In the second and third surveys, we used a simple questionnaire on only asthmatic symptoms and changes in the residential environment in the past year. These questionnaires were filled out by either parent. All incomplete questionnaires, accompanied by a request for completion, were returned to the subjects. We obtained informed consent with signatures by the children’s parents in the questionnaires.

The definitions of asthma and wheeze within the questionnaire were the same as in our previous study.^17^ Briefly, asthma was defined as “two or more episodes of wheezing accompanied by dyspnea that had ever been given the diagnosis of asthma by a physician” and “the occurrence of asthmatic attacks or the need for any medication for asthma during the past two years.” For children who had no symptom of asthma, wheeze was a positive response to the question “Has your child had wheezing or whistling in the chest during the last 12 months?”. Children who had been diagnosed as having eczema, atopy, allergic rhinitis, or pollinosis by a physician, or who had received hyposensitization therapy, were considered to have a history of allergic diseases.

For children who had neither current symptom nor past history of asthma in the first survey, the new onset of asthmatic symptoms in the second, third, and fourth surveys was evaluated. For children who had no symptom of asthma or wheeze in the first survey, the incidence of wheeze in the fourth survey was also evaluated. The definitions of asthma and wheeze were consistent across the four surveys.

Children who had resided at the present address less than three years before the first survey were excluded from the analysis. For children from the six urban schools, the distance between their homes and the trunk roads was measured on maps. Subjects were then classified into children who lived <50 m from the edge of trunk roads (roadside area) and children who lived 50+ m from the roads (non-roadside area). The number of children living in roadside areas ranged from 10 to 44 per school. Then, we compared the prevalence and incidence of respiratory symptoms for roadside, non-roadside, and rural areas. The trends in these rates for roadside > non-roadside > rural areas were evaluated in relation to the concentration of air pollution in each area.

The prevalence of respiratory symptoms in the first survey was compared in relation to sex, maternal smoking habits, structure of the house, type of heating appliance, and location of the home. For children from whom questionnaires were available in all four surveys, the prevalence of asthma and wheeze in each survey was compared in relation to the location of the home separately by sex.

The incidence rate of asthma or wheeze during the follow-up period was compared in relation to the location of the home among children who had no symptoms in the first survey. Logistic regression models were used to evaluate the effects of various factors on the incidence of asthma or wheeze separately by sex. As independent variables the models included the location of the home, school grade, history of allergic diseases, respiratory diseases before the age of two years, feeding method in infancy, parental history of allergic diseases, maternal smoking habits, structure of the house, and type of heating appliance. All analyses were conducted using SAS^®^ software (Version 6, SAS Institute, Inc., Cary, NC).

## RESULTS

**Figure 2.**
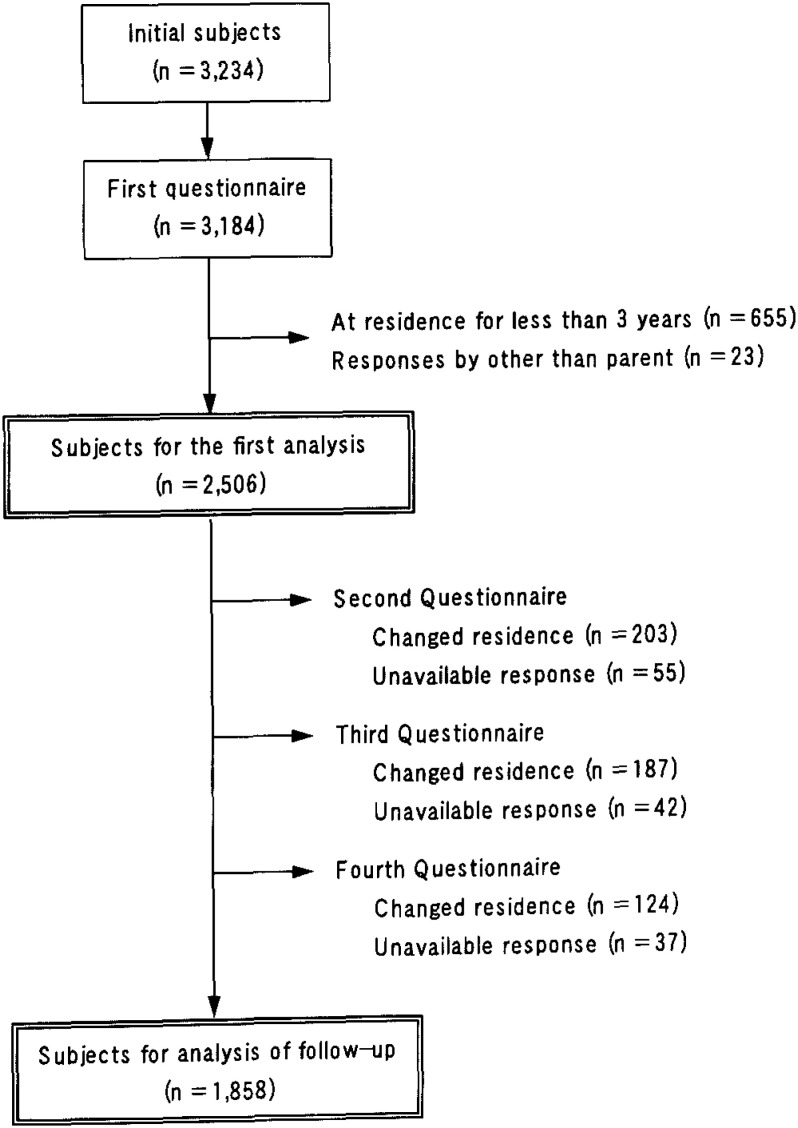
Schematic representation of the selection process and sample attrition.

Completed respiratory symptom questionnaires were obtained for 3,184 children (98.5%) in the target population on the first survey. The sample size for the first analysis was comprised of 2,506 children (77.5%). Excluded were 655 children whose residence had changed within the three years prior to the survey and 23 children whose questionnaire had been answered by someone other than a parent (Figure 2).

Responses to the questionnaire were not available for 648 children in the second to fourth surveys, primarily because of changed residence. Thus, the final sample for follow-up analysis was comprised of 1,858 children (74.1% of the subjects in the first survey). The characteristics of the study subjects followed for three years and subjects not followed are shown in Table 3. The percentage of children who were followed was highest in the rural areas, although there was no difference between roadside and non-roadside areas in urban communities.

**Table 3.** Initial characteristics of the study subjects followed or not-followed for 3 years.

Characteristics	Follow-up (n = 1,858) (%)	No follow-up (n = 648) (%)	p-value
Sex			
Male	51.2	48.1	0.183
Female	48.8	51.9	
Asthma	6.6	8.3	0.143
Wheeze	5.2	6.0	0.440
History of allergic diseases	54.6	49.7	0.030
Respiratory diseases before 2 years of age	10.7	10.3	0.792
Breast feeding in infancy	27.7	34.4	0.001
Parental history of allergic diseases	45.3	44.8	0.822
Maternal smoking habits	10.8	14.0	0.025
House of steel or reinforced concrete	31.1	42.9	< 0.001
Use of unvented heater in winter	63.1	67.0	0.075
Areas			
Urban areas			
0-49 m from trunk roads	6.7	7.9	0.002
≥ 50 m from trunk roads	48.2	55.1	
Rural areas	45.1	37.0	

The prevalence rates of respiratory symptoms in the first survey are shown in Table 4. In comparison with children living in wooden houses, the prevalence of bronchitis among both boys and girls and of asthma among girls was significantly higher in children living in steel or reinforced concrete houses. The prevalence of respiratory symptoms did not significantly differ in relation to maternal smoking and type of heating appliance among either boys or girls. The prevalence of cough or phlegm was very low and not related to any of these variables.

**Table 4.** Prevalence of respiratory symptoms in the first survey in relation to environmental factors.

	N	History of allergic diseases	Bronchitis ever	Chronic cough	Chronic phlegm	Asthma	Wheeze
Males							
Maternal smoking habits							
Yes	156	48.1	23.1	1.3	1.9	7.7	8.3
No	1,107	55.6	21.3	1.3	1.1	7.0	5.4
p-value		0.091	0.692	0.716	0.609	0.898	0.202
Structure of house							
Steel or reinforced concrete	404	57.4	26.7	2.0	1.5	7.9	7.7
Wood	859	53.4	19.1	0.9	1.0	6.8	4.9
p-value		0.205	0.003	0.199	0.696	0.525	0.063
Type of heating appliance							
Unvented	804	53.4	21.5	1.6	1.2	6.7	6.0
Vented	459	57.1	21.6	0.7	1.1	7.8	5.4
p-value		0.223	0.960	0.226	0.979	0.525	0.796
Areas							
0-49 m from trunk roads	82	62.2	25.6*	0.0	0.0	8.5	8.5
≥ 50 m from trunk roads	623	57.1*	25.5**	1.3	1.3	7.9	7.1*
Rural areas	558	50.9	16.5	1.4	1.3	6.1	3.9
p-value		0.010	< 0.001 ^†^	0.555	0.590	0.210^†^	0.012^†^

Females							
Maternal smoking habits							
Yes	135	51.9	22.2	3.7	3.7	8.9	7.4
No	1,108	52.0	20.7	2.1	1.5	6.8	4.8
p-value		0.951	0.758	0.370	0.145	0.464	0.269
Structure of house							
Steel or reinforced concrete	451	54.5	24.6	1.8	1.3	9.3	4.9
Wood	792	50.5	18.7	2.5	2.0	5.7	5.2
p-value		0.190	0.016	0.509	0.507	0.022	0.923
Type of heating appliance							
Unvented	802	53.7	21.1	1.9	1.7	7.2	5.6
Vented	441	48.8	20.4	2.9	1.8	6.6	4.1
p-value		0.104	0.839	0.305	0.891	0.751	0.298
Areas							
0-49 m from trunk roads	93	53.8	28.0**	1.1	1.1	8.6	5.4
≥ 50 m from trunk roads	630	54.0	23.3**	1.9	1.7	87**	5.7
Rural areas	520	49.2	16.5	2.9	1.9	4.6	4.2
p-value		0.260	0.001 ^†^	0.392	0.848	0.020	0.516

* p < 0.05, ** p < 0.01, compared with children living in rural areas.

^†^ p-value for the trend in the rates for roadside > non-roadside > rural area.

Among boys, the prevalence rates of allergic diseases, bronchitis, and wheeze were highest in roadside areas and lowest in rural areas. The prevalence of asthma was highest in roadside areas, but the difference among areas was not significant. Among girls, the prevalence rate of bronchitis was highest in roadside areas. The prevalence rates of allergic diseases, asthma, and wheeze were lowest in rural areas, although there were no differences between roadside and non-roadside areas.

The city-specific prevalence rates of respiratory symptoms are shown in Table 5. The prevalence rates of bronchitis were higher among both boys and girls living in Chiba, Funabashi, and Kashiwa. However, the prevalence of other symptoms did not significantly differ in relation to cities.

**Table 5.** Prevalence of respiratory symptoms in the first survey in relation to study communities.

	N	History of allergic diseases	Bronchitis ever	Chronic cough	Chronic phlegm	Asthma	Wheeze
Males							
Chiba	112	58.9	30.4	1.8	0.0	10.7	7.1
Funabashi	207	55.1	27.5	0.5	1.0	6.8	7.7
Kashiwa	165	60.6	24.8	1.8	1.8	7.3	5.5
Ichikawa	221	57.5	21.7	0.9	1.4	8.1	8.1
Ichihara	157	52.2	15.3	1.9	1.3	5.1	4.5
Tateyama	179	47.5	18.4	1.1	1.1	7.3	4.5
Mobara	93	52.7	19.4	2.2	1.1	5.4	4.3
Kisarazu	129	52.7	13.2	0.8	1.6	6.2	2.3
p-value		0.305	0.004	0.859	0.948	0.762	0.299

Females							
Chiba	127	55.1	30.7	3.9	3.9	8.7	4.7
Funabashi	219	52.1	25.6	0.5	0.0	10.0	6.8
Kashiwa	176	54.0	26.7	1.1	2.3	7.4	5.1
Ichikawa	201	55.2	15.4	2.5	1.5	8.5	5.5
Ichihara	133	48.1	16.5	3.8	3.8	5.3	3.8
Tateyama	157	48.4	19.1	3.8	2.5	5.1	5.1
Mobara	85	50.6	20.0	0.0	0.0	4.7	4.7
Kisarazu	145	50.3	11.7	2.8	0.7	3.4	3.4
p-value		0.841	< 0.001	0.132	0.055	0.229	0.900

**Figure 3.**
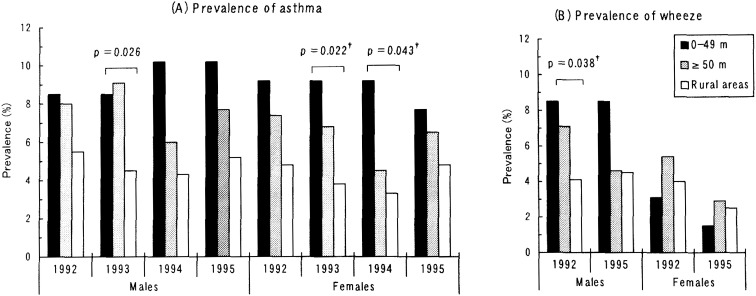
Prevalence rates of asthma and wheeze in each survey, in relation to location of homes, ^†^ p-value for the trend in the rates for roadside > non-roadside > rural area.

The prevalence rates of asthma and wheeze in each survey by location of homes are shown in Figure 3. Over the four surveys, the prevalence of asthma was lowest in rural areas among both boys and girls. The prevalence of asthma in the second survey was significantly lower among boys in rural areas than in other areas. Among girls, testing for trends in the second and third surveys showed that the prevalence of asthma increased significantly in the order of roadside > non-roadside > rural areas. The prevalence of wheeze was highest among boys in roadside areas in the first and fourth surveys, but there was no difference in relation to the location of the home among girls.

**Figure 4.**
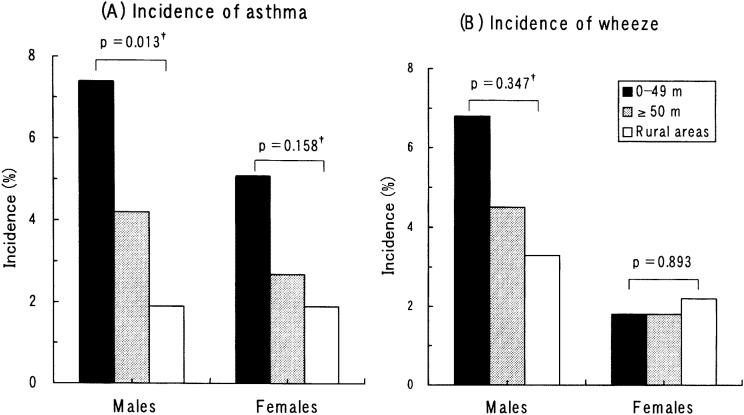
Incidence rates of asthma and wheeze for the follow-up period, in relation to location of homes. ^†^ p-value for the trend in the rates for roadside > non-roadside > rural area.

The incidence rates of asthma and wheeze in the follow-up period were evaluated among children who had no symptoms in the first survey (Figure 4). Among both boys and girls, asthmatic symptoms tended to in the order of roadside > non-roadside > rural areas. The trend was significant among boys. The incidence of wheeze did not differ in relation to the location of the home among either boys or girls.

The logistic regression models examined the effects of various factors on the incidence of asthma and wheeze (Table 6). Among boys, the adjusted odds ratios (OR) of the incidence of asthma were 3.77 for roadside and 1.99 for non-roadside areas relative to rural areas. Among girls, the values were 4.03 and 1.74, respectively. The value for boys in roadside areas was significantly different from one. The incidence of asthma significantly increased in relation to a history of allergic diseases among both boys and girls and in relation to parental history of allergic diseases among only boys. Among either boys or girls, the location of the home was not related to the incidence of wheeze. The factors that significantly impacted on the incidence of wheeze were school grade, a history of allergic diseases and respiratory disease under two years of age among only boys. The effects of breast-feeding in infancy, maternal smoking habits, structure of the house, and type of heating appliance were not significant in relation to either asthma or wheeze.

**Table 6.** Odds ratios (OR) and 95% confidence intervals (95% CI)* for various risk factors on the incidence of asthma or wheeze for the follow-up period.

Factors	Asthma			Wheeze		
	
	OR	(95% CI)	p-value	OR	(95% CI)	p-value
Males						
Areas						
Rural areas	1			1		
≥50 m from trunk roads	1.99	(0.79-4.99)	0.144	1.02	(0.44-2.36)	0.971
0-49 m from trunk roads	3.77	(1.00-14.16)	0.049	1.35	(0.34-5.32)	0.670
School grade, 1 grade increase	1.13	(0.70-1.83)	0.624	0.55	(0.34-0.89)	0.014
History of allergic diseases	2.95	(1.17-7.46)	0.022	3.26	(1.30-8.17)	0.012
Respiratory diseases before 2 years of age	1.85	(0.70-4.92)	0.216	2.40	(1.01-5.72)	0.048
Breast feeding in infancy	1.42	(0.64-3.18)	0.390	0.47	(0.19-1.20)	0.113
Parental history of allergic diseases	2.82	(1.21-6.55)	0.016	1.71	(0.81-3.63)	0.163
Maternal smoking habits	1.74	(0.62-4.91)	0.294	0.45	(0.10-1.96)	0.285
House of steel or reinforced concrete	0.92	(0.39-2.16)	0.843	1.05	(0.45-2.47)	0.909
Use of unvented heater in winter	1.47	(0.64-3.40)	0.364	0.65	(0.31-1.36)	0.251

Females						
Areas						
Rural areas	1			1		
≥50 m from trunk roads	1.74	(0.63-4.81)	0.286	0.79	(0.26-2.39)	0.673
0-49 m from trunk roads	4.03	(0.90-17.96)	0.068	0.76	(0.08-7.00)	0.812
School grade, 1 grade increase	0.94	(0.54-1.64)	0.827	0.98	(0.53-1.82)	0.947
History of allergic diseases	6.03	(1.74-20.83)	0.005	3.18	(0.99-10.20)	0.052
Respiratory diseases before 2 years of age	2.08	(0.64-6.73)	0.222	2.10	(0.57-7.80)	0.267
Breast feeding in infancy	0.60	(0.20-1.83)	0.369	2.13	(0.77-5.90)	0.146
Parental history of allergic diseases	1.20	(0.49-2.94)	0.693	0.47	(0.16-1.39)	0.170
Maternal smoking habits ^†^	2.15	(0.64-7.21)	0.218	-		
House of steel or reinforced concrete	0.40	(0.13-1.20)	0.102	0.64	(0.18-2.27)	0.486
Use of unvented heater in winter	0.77	(0.30-2.00)	0.596	0.60	(0.22-1.68)	0.333

* Odds ratios adjusted for all variables using the logistic regression model.

^†^ Not included into the analysis of wheeze among females, because wheeze had occurred in only one girl who had a smoking mother.

## DISCUSSION

In our study population, the prevalence and incidence of asthma increased among children who lived near trunk roads with heavy traffic. The incidence of asthma was significantly higher among boys who lived in roadside areas than among those in rural areas, even after adjustment for potential confounding factors such as a history of allergic diseases and indoor environment. This result showed that asthma tended to develop in association with the level of air pollution.

The prevalence of asthma has been increasing in many countries.^19^^,^^20^ Air pollutants, such as automobile exhaust, have been considered as one of the potential risk factors of wheezing bronchitis or asthma.^16^^,^^21^ Exposure to ambient air pollution was shown to aggravate symptoms of asthma.^10^^-^^11^ However, there have been few prospective cohort studies that estimated the incidence of asthma or wheeze. Neas et al.^22^ conducted a three-year cohort study to examine the effect of NO_2_ on the annual cumulative incidence of respiratory symptoms during the prior year. However, they did not report rates of newly developed asthma. In a study of children living in 8 areas of Japan for 5 years, the incidence of asthma tended to increase in areas with an annual mean NO_2_ concentration > 0.03 ppm, although the effects of confounding factors, including allergy and indoor air pollution, were not considered.^23^

In this study, the prevalence and cumulative incidence of asthma and wheeze were examined. Respiratory symptoms were evaluated by a standardized questionnaire in the first and fourth survey and a simple questionnaire on only asthmatic symptoms in the second and third surveys because the latter was easy to complete. The difference in questionnaires might lead to potential bias in responses.^24^ However, the two questionnaires included the same questions about asthmatic symptoms, and the definitions of asthma were unified. Therefore, we have considered that misclassification due to the difference in questionnaires was minimal.

The concentrations of air pollutants were higher in urban than in rural communities. Our study districts in urban communities were intersected by trunk roads, and the concentrations of air pollutants were especially high in roadside areas. Concentrations of air pollutants were reported to be highest at the edge of trunk roads and decreased according to distance from the roadside.^1^^,^^3^ In the present study, children who lived < 50 m from the edge of a road were classified as roadside subjects. In using nearly the same communities as in the present study, the indoor NO_2_ concentrations during the non-heating period were reported to be higher in homes < 50 m from the edge of roads than in homes located in other areas.^2^ Subjects who live near trunk roads should be exposed to high concentrations of air pollutants primarily derived from automobile exhaust.

Diesel exhaust has been shown to enhance allergic airway inflammation and hyperresponsiveness in mice.^25^ Occupational exposure to diesel exhaust was demonstrated to lead to the development of asthma among railroad workers.^8^ Consequently, there has been concern about the effects of diesel exhaust on the health of individuals who reside near trunk roads. Nitta et al.^3^ reported a high prevalence of respiratory symptoms in female adults who lived along major roads in Tokyo, Japan. Several European studies have shown that air pollution from heavy traffic was associated with the prevalence of asthma and respiratory symptoms among children living in urban areas.^14^^,^^26^^-^^29^ Edward et al.^30^ showed a high risk of hospital admission for asthma among children who lived near major roads with high traffic flow. The present study also showed a high prevalence and incidence of asthma among children who lived in roadside areas, suggesting the effect of automobile exhaust on asthma among children.

Living in roadside areas was also significantly associated with a high prevalence of asthma among girls and of wheeze among boys. Thus, the prevalence rates of respiratory symptoms in relation to the location of homes were different between boys and girls. Others have reported that girls are more susceptible to air pollution than boys.^12^^,^^28^^,^^29^ However, our previous study showed that pulmonary function was associated with air pollution among boys.^31^ Constitutional factors linked to sex may be of importance in the effects of air pollution on respiratory symptoms or diseases.

A history of allergic diseases in the subjects or their parents was also associated with the incidence of asthma or wheeze. Half of the subjects had a history of allergic diseases, among which atopy and allergic rhinitis were dominant. Atopic status of the subjects and their parents was shown to influence the incidence of asthma and wheeze during adulthood.^32^^,^^33^ No increase in allergic diseases was observed with increasing levels of air pollution in the International Study of Asthma and Allergy in Childhood (ISAAC).^15^^,^^20^ The comparisons between eastern and western European populations showed a lower prevalence of allergic diseases in eastern areas with high levels of sulfur oxides and suspended particles.^34^^,^^35^ A recent report from Germany showed that traffic-related air pollution increased the prevalence of allergic diseases,^36^ although the authors found no effect of either sulfur oxides or suspended particles on the development of allergies.^37^

The present study showed an increased prevalence of allergic diseases in roadside areas only among boys. Diesel exhaust particles have been implicated in human allergic diseases by enhancing the production of IgE antibodies.^38^^,^^39^ We reported previously that serum hyaluronate levels were increased in children who lived near busy roads compared with those who lived farther away, but only among children in whom serum IgE levels had been elevated.^5^ Hyaluronate is considered to serve as a possible biomarker for lung diseases, and our results suggest that children with an atopic predisposition may be susceptible to the effects of automobile exhaust.

There was no difference in the incidence of wheeze in relation to the location of homes. The effects of a history of allergic diseases and respiratory diseases before 2 years of age on wheeze were too great to separate out the effect of automobile exhaust. Weiss et al.^40^ reported a possible association of respiratory illness in early life with increased levels of airway responsiveness. Henderson et al.^41^ showed that a history of respiratory disease before 2 years of age was associated with recurrent wheezing in school-age children.

To evaluate the effects of air pollution on health, the potential involvement of indoor air pollution should be considered.^42^ In the present study, neither prevalence nor incidence of any respiratory symptoms was associated with the type of heater that is usually considered as a major source of indoor air pollution in Japan. Maternal smoking is considered as an important risk factor for childhood asthma and wheezing.^43^ However, Chinn and Rona^44^ found that exposure to environmental tobacco smoke was associated with wheezing but not with diagnosed asthma. In the present study, maternal smoking was not related to any respiratory symptom in children. This may be because the number of children whose mother smoked was small. The prevalence of bronchitis was higher among children living in steel or reinforced concrete houses than in wooden houses. The airtightness of the dwelling may be involved with respiratory symptoms, although there was no association between the structure of the house and the incidence of asthma or wheeze.

In cohort studies, a substantial number of subjects may be inevitably lost to follow-up primarily because of a change in residence. Forastiere et al.^45^ obtained responses from 84.6% of the primary subjects after a 3.5-year interval in a cohort of children. We were able to follow up 1,858 children (74.1% of the subjects in the first survey) for three years, which satisfied the purpose of our study. The incidence and prognosis of asthma in childhood should be further evaluated in a longitudinal study for many more years. Oosterlee et al.^28^ reported that families with asthmatic children had lived near busy roads for shorter times than families with nonasthmatic children. It may be that parents of children with respiratory symptoms preferred not to live along busy roads. Such a selective migration in relation to asthmatic symptoms in children should lead to underestimation of the risk of living in roadside areas, although the housing situation is not always similar in Japan and European countries. However, the incidence in a cohort study should be less influenced by selective migration than the prevalence in a cross-sectional study.

In the present study, subjects were categorized according to the distance of their homes from trunk roads. Daytime average traffic flow of these roads ranged from 37,000 to 83,000 vehicles every 12 hours, and heavy vehicles with diesel engines accounted for 28.2% of vehicles. In recent years, fine particles in diesel exhaust have been reported to be associated strongly with respiratory symptoms.^13^^,^^46^ However, in Japan, atmospheric concentrations of fine particles have been rarely measured. Exposure to air pollutants including diesel exhaust in roadside areas should be further evaluated.

In conclusion, this study showed that the prevalence and incidence of asthma increased among children who live near trunk roads with heavy traffic. The risk of the incidence of asthma remained significant among boys who lived in roadside areas, even after adjustment for the effects of allergies that have an important role in the development of asthma in childhood. These findings suggest that air pollution primarily derived from automobile exhaust may be particularly important for the development of asthma among children living near trunk roads with heavy traffic. The incidence and prognosis of respiratory symptoms should be further evaluated in a longitudinal study for many more years.
